# Supplementary figures and images for: Learning the Effect of Registration Hyperparameters with HyperMorph
Source: J Mach Learn Biomed Imaging. Author manuscript; Available in PMC 2022 Sep 21. (PMC9491317)

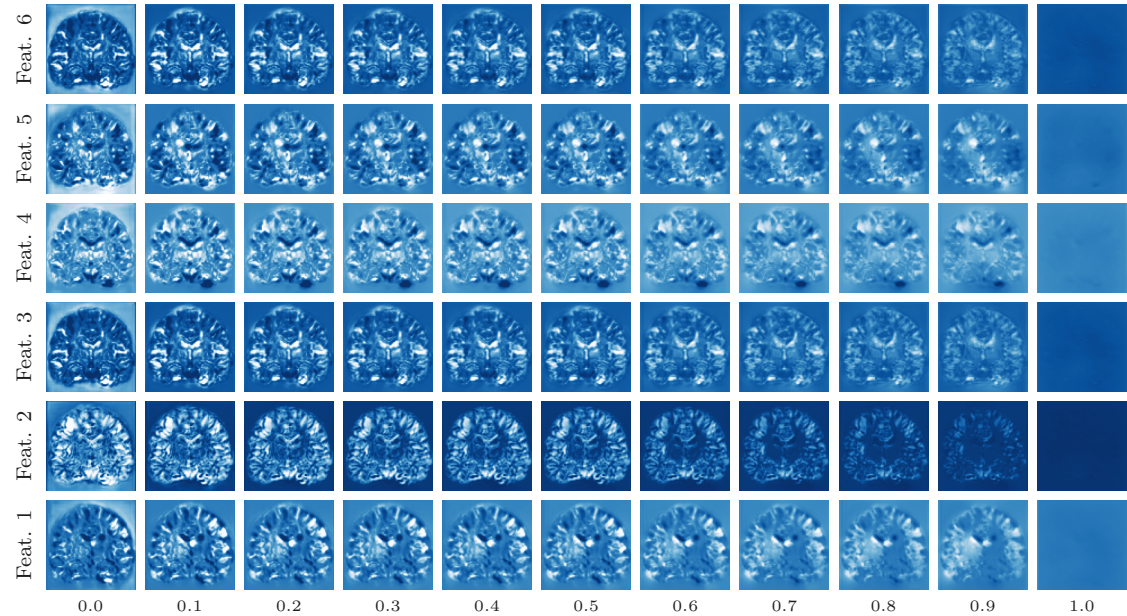

$$\lambda (\mathcal{L}_{sim} = MSE)$$

Supplement: Figure S2 — Changes in feature activations of the final HyperMorph U-Net layer across different values for λ. [file NIHMS1835691-supplement-Figure_S2.pdf]

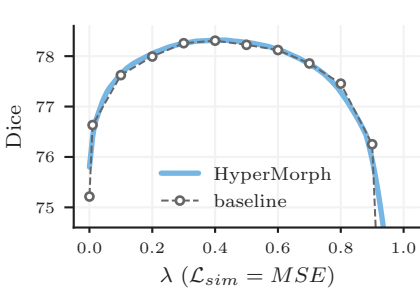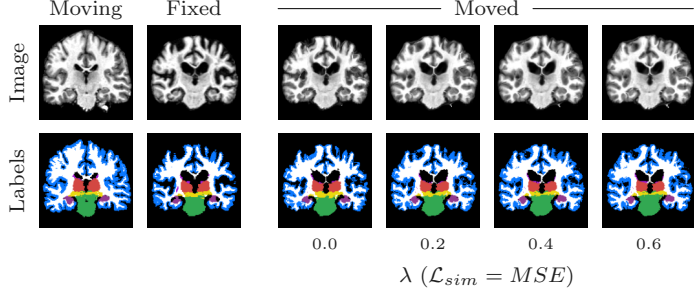

Supplement: Figure S1 — Left: mean Dice scores achieved by a single HyperMorph model and baselines evaluated on the held-out, manually-labeled Buckner40 dataset. Right: image and label-based qualitative changes in HyperMorph alignment across different regularization weights for a given subject pair. [file NIHMS1835691-supplement-Figure_S1.pdf]
